# Metformin promotes apoptosis in hepatocellular carcinoma through the CEBPD-induced autophagy pathway

**DOI:** 10.18632/oncotarget.14640

**Published:** 2017-01-13

**Authors:** Hsin-Hwa Tsai, Hong-Yue Lai, Yueh-Chiu Chen, Chien-Feng Li, Huei-Sheng Huang, Hsiao-Sheng Liu, Yau-Sheng Tsai, Ju-Ming Wang

**Affiliations:** ^1^ Institute of Bioinformatics and Biosignal Transduction, National Cheng Kung University, Tainan, Taiwan; ^2^ Institute of Basic Medical Sciences, National Cheng Kung University, Tainan, Taiwan; ^3^ Department of Pharmacology, National Cheng Kung University, Tainan, Taiwan; ^4^ Department of Pathology, Chi-Mei Medical Center, Tainan, Taiwan; ^5^ Department of Medical Laboratory Science and Biotechnology, National Cheng Kung University, Tainan, Taiwan; ^6^ Department of Microbiology and Immunology, National Cheng Kung University, Tainan, Taiwan; ^7^ Institute of Clinical Medicine, National Cheng Kung University, Tainan, Taiwan; ^8^ Graduate Institute of Medical Sciences, Taipei Medical University, Taipei, Taiwan

**Keywords:** HCC, autophagy, apoptosis, CEBPD, metformin

## Abstract

Metformin, as an AMP-activated protein kinase (AMPK) activator, can activate autophagy. A study showed that metformin decreased the risk of hepatocellular carcinoma (HCC) in diabetic patients. However, the detailed mechanism in the metformin-mediated anticancer effect remains an open question. Transcription factor CCAAT/enhancer-binding protein delta (CEBPD) has been suggested to serve as a tumor suppressor and is responsive to multiple anticancer drugs in HCC. In this study, we found that CEBPD and autophagy are involved in metformin-induced cell apoptosis in Huh7 cells. The underlying mechanisms in this process included a reduction in Src-mediated CEBPD protein degradation and an increase in CEBPD-regulated *LC3B* and *ATG3* gene transcription under metformin treatment. We also found that AMPK is involved in metformin-induced CEBPD expression. Combined treatment with metformin and rapamycin can enhance autophagic cell death through the AMPK-dependent and AMPK-independent pathway, respectively. Taken together, we provide a new insight and therapeutic approach by targeting autophagy in the treatment of HCC.

## INTRODUCTION

Autophagy is a conserved intracellular degradation process in which cellular organelles are degraded by lysosomes and recycled to replenish cells with energy in stress environments. As an essential regulator of cellular homeostasis and adaptation of stresses, autophagy plays an important role in carcinogenesis including hepatocellular carcinoma (HCC). Although the role of autophagy in hepatocarcinogenesis remains controversial [1, 2], studies have demonstrated the crucial role of autophagy in liver disease. Therefore, understanding the details of the involvement of autophagy in HCC is crucial to help facilitate the development of future therapeutic approaches to HCC.

Autophagy related genes (ATGs) are vital for phagophore formation, and microtubule-associated protein light chain 3 (LC3B, also known as ATG8) is one of the most important phagophore components. Several specific substrates are preferentially degraded by autophagy. Of these, the best-known protein is p62 [also known as sequestosome 1 (*SQSTM1*)]. In HCC, p62 expression increases, and the autophagy response is impaired [3]. In addition, enhanced LC3B can be a strong prognosis factor [4]. However, the regulation of ATGs and their involvement in HCC cancer cells remain largely uncharacterized.

Metformin, a biguanide class of oral hypoglycemic agents, is the first line drug for the treatment of type 2 diabetes mellitus [5]. Epidemiological studies reported that metformin can reduce the risk of HCC occurrence [6–8]. Metformin also induces G1 phase arrest and apoptosis in HCC [9, 10]. Metformin is an AMP-activated protein kinase (AMPK) activator and can activate autophagy via a reduction in the mammalian target of rapamycin (mTOR) signaling pathway [11]. Previous studies demonstrated that metformin-induced autophagy performs a proapoptotic role in melanoma and lymphoma [11, 12]. However, the detailed mechanisms linking autophagy and apoptosis in the metformin-mediated anticancer effect on HCC remain an open question.

CCAAT/enhancer-binding protein delta (CEBPD) is a transcription factor that belongs to the CCAAT/enhancer-binding protein family. Several studies demonstrated that CEBPD expression is downregulated in breast cancer [13], leukemia [14], cervical cancer [15], and hepatocellular carcinoma [16]. CEBPD is thought to be a potent tumor suppressor, as its overexpression can result in the death of cancer cells. For instance, CEBPD can upregulate proapoptotic genes, including PPARG2 and GADD153, in cervical cancer [15] and caspase-8 and caspase-3 in prostate cancer [17]. Thus, activating CEBPD expression in cancer cells could be a strategy for cancer therapy.

## RESULTS

### Autophagy involves in metformin-induced cell apoptosis in Huh7 cells

**Figure 1:**
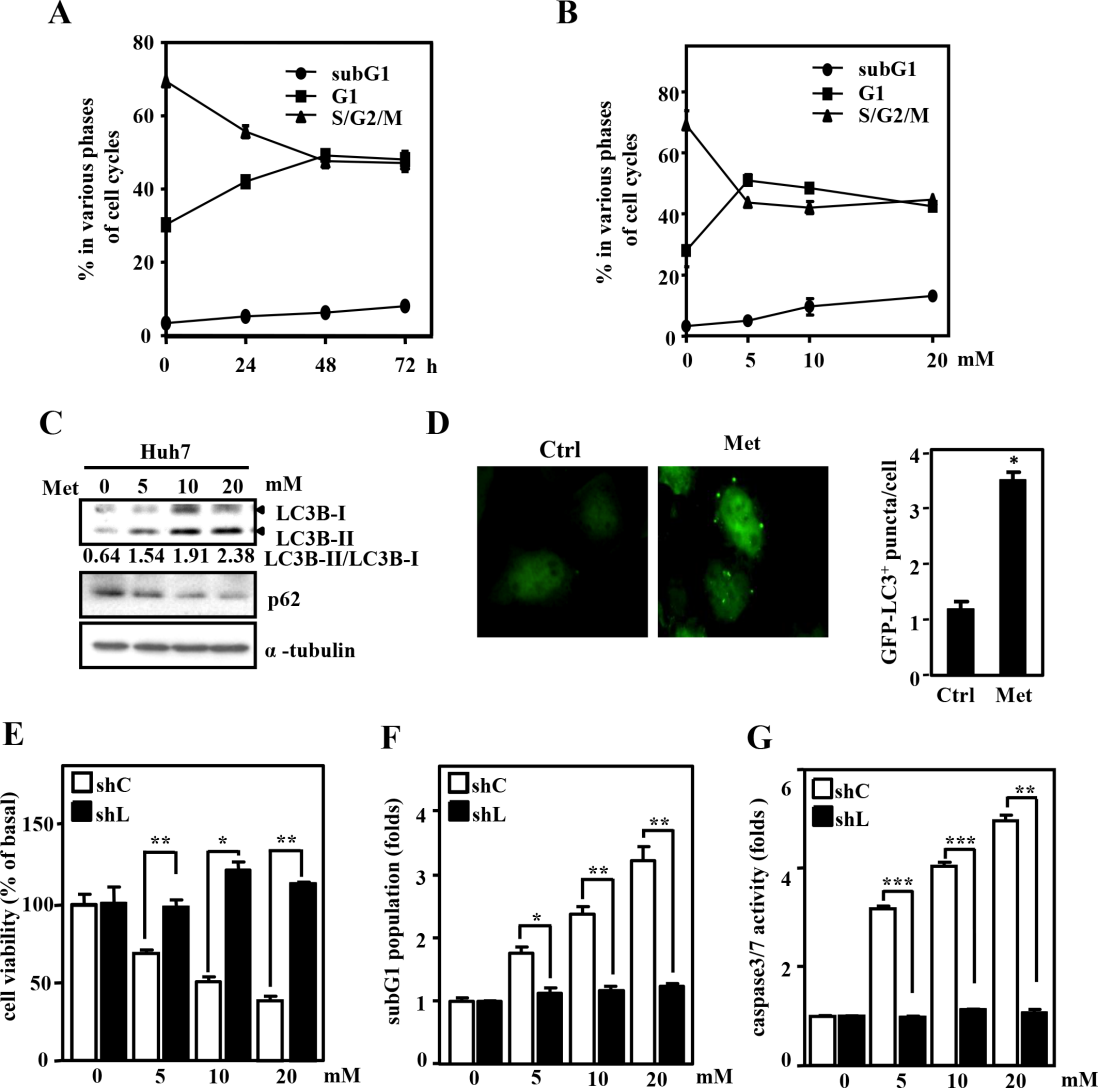
Autophagy plays a pro-apoptotic role in metformin-treated Huh7 cells (**A**) Huh7 cells were treated with metformin (Met, 5 mM) for 24, 48 and 72 h or (**B**) treated with Met (5, 10 and 20 mM) for 48 h. Experimental cells were collected at the indicated concentrations and time points, stained with propidium iodide, and analyzed by flow cytometry. (**C**) Huh7 cells were treated with Met (5 mM) for 6 h and harvested for Western blot analyses. (**D**) Huh7 cells were transfected with GFP-LC3B expression vectors and then treated with or without Met (5 mM) for 6 h. The numbers of LC3B puncta were evaluated under a fluorescence microscope. (**E**) Huh7 cells were infected with lentiviruses encoding shLacZ (shC) or shLC3B (shL) for 3 days. The cell viability of infected experimental cells was measured by MTT assays after 48 h of Met treatment at the indicated concentrations. (**F**) The apoptotic activity of these experimental cells was analyzed by flow cytometry PI staining. (**G**) The caspase-3/7 activity of these experimental cells was detected by CellEvent caspase-3/7 green detection reagent.

To dissect the details of metformin function in liver cancer cells, we first assessed the survival effect on Huh7 cells. The results showed that the growth arrest and apoptosis of Huh7 cells were coordinately increased following the metformin treatment (Figure 1A and Supplementary Figure 1A). Meanwhile, lower doses of metformin induced growth arrest, indicating that apoptosis should follow the induction of growth arrest in response to higher doses of metformin (Figure 1B and Supplementary Figure 1B). We next assessed whether the activation of autophagy was also responsive to metformin. To address this issue, characteristic hallmarks of autophagy were examined in Huh7 cells. We found that the LC3B-II/LC3B-I ratio was increased and p62 expression was reduced in metformin-treated Huh7 cells (Figure 1C). In addition, metformin increased the numbers of LC3B puncta in Huh7 cells that were transfected with GFP-LC3B expression vectors (Figure 1D). Activation of autophagy can lead to either cell survival or death [18]. Currently, the role of autophagy in metformin-mediated apoptosis of liver cancer cells is unknown. To address this, LC3B was knocked down by lentiviruses with shLC3B. An MTT assay showed that LC3B knockdown restored metformin-inhibited cell viability in Huh7 cells (Figure 1E). Furthermore, LC3B knockdown attenuated metformin-induced cell apoptosis, as demonstrated by flow cytometry propidium iodide (PI) staining and TUNEL assay, and repressed metformin-induced caspase-3/7 activation in Huh7 cells (Figure 1F, 1G and Supplementary Figure 1C). This result was confirmed by treatment with an autophagy inhibitor (chloroquine, CQ), which inhibits both the fusion of autophagosomes with lysosomes and lysosomal protein degradation [19]. Treatment with CQ also restored metformin-inhibited cell viability and repressed metformin-induced caspase-3/7 activation in Huh7 cells (Supplementary Figure 2A and 2B). Taken together, these results suggest that autophagy plays a pro-apoptotic role in the metformin-mediated anticancer effect in Huh7 cells.

### CEBPD participates in metformin-mediated anticancer effects in Huh7 cells

**Figure 2:**
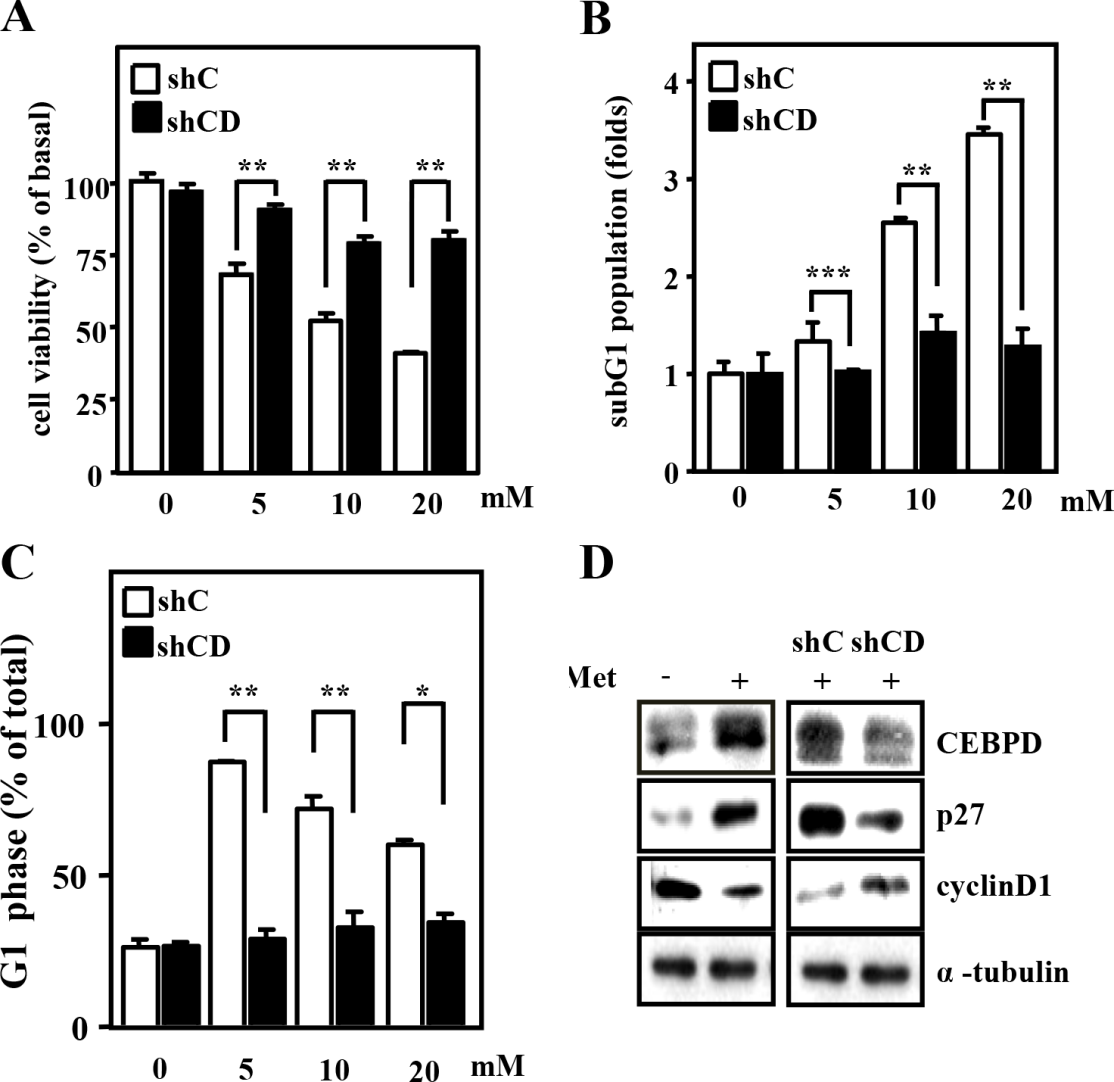
CEBPD contributes to metformin-induced cell cycle arrest and cell apoptosis in Huh7 cells (**A**) Huh7 cells were infected with lentiviruses encoding shLacZ (shC) or shCEBPD (shCD) for 3 days. After treating with metformin (Met) at the indicated concentrations (5, 10 and 20 mM) for 48 h, the cell viability of infected experimental cells was measured by MTT assays. (**B**) The apoptotic activity of the experimental cells was analyzed by flow cytometry PI staining. (**C**) The G1 phase of these experimental cells was analyzed by flow cytometry. (**D**) Huh7 cells were infected with lentiviruses encoding shLacZ (shC) or shCEBPD (shCD) for 3 days. After treating with or without Met (5 mM) for 6 h, the lysates were harvested for Western blot analyses.

Previous results showed that CEBPD expression was upregulated by several clinical anticancer drugs in Huh7 and HepG2 cells [15]. Here, we found that CEBPD expression was also induced by metformin in liver cancer cells (Supplementary Figure 3). To verify whether CEBPD contributes to the metformin-induced anticancer effects, a loss-of-function assay in which CEBPD was knocked down by shRNA was performed. Loss of CEBPD attenuated metformin-induced cytotoxic activity in Huh7 cells (Figure 2A), indicating that CEBPD contributed to metformin-inhibited cell viability. Moreover, the processes of apoptosis (Figure 2B and Supplementary Figure 1D) and G1 arrest (Figure 2C) contributed to CEBPD-mediated antiproliferation activity upon metformin treatment. The upregulation and downregulation of the CEBPD responsive genes cyclin D1 [20] and p27 [21], respectively, were observed following CEBPD attenuation upon metformin treatment (Figure 2D).

### Metformin induces CEBPD protein stabilization via reduced Src activity

**Figure 3:**
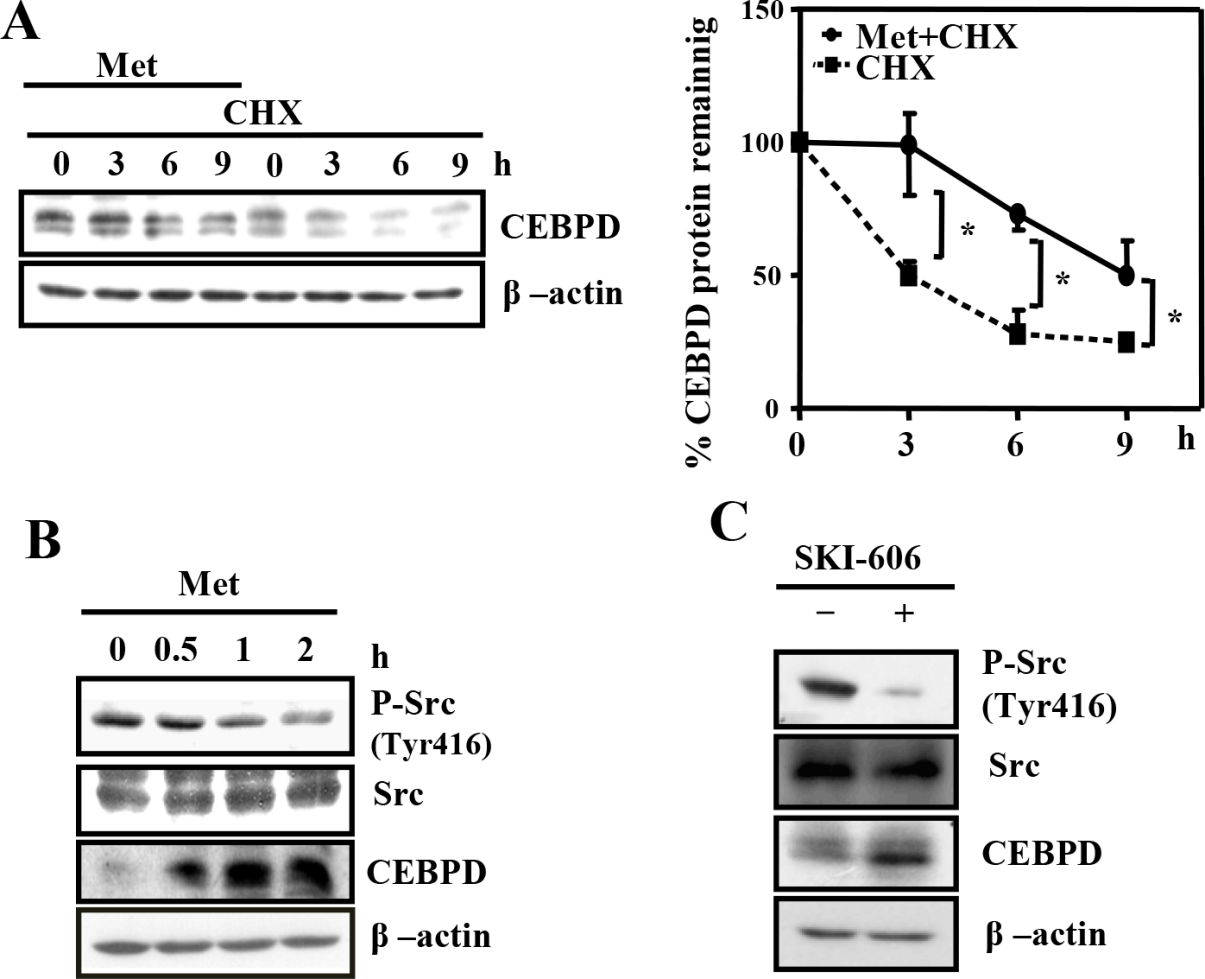
Metformin stabilizes CEBPD protein through reduced Src activity in Huh7 cells (**A**) Huh7 cells were pretreated with or without Met (5 mM) for 2 h and then treated with cyclohexamide (CHX, 10 μg/ml) to inhibit *de novo* synthetic proteins. Protein lysates were extracted at the indicated time points, and the remaining proteins were analyzed by Western blotting. (**B**) Huh7 cells were treated with Met (5 mM) at indicated time points or (**C**) treated with SKI-606 for 24 h. The experimental cell lysates were harvested for Western blot analyses.

Several studies indicated that CEBPD abundance can be regulated in a transcription-dependent [16, 22] or -independent manner [23]. We found that metformin had no effect on the regulation of CEBPD transcription (Supplementary Figure 4A and 4B) but affected its protein stability (Figure 3A). We next tested whether Src kinase-increased proteasome degradation [23] contributed to metformin-induced attenuation of CEBPD protein degradation. Metformin inhibited Src activity and enhanced CEBPD expression (Figure 3B). Moreover, the inhibition of Src kinase by a Src inhibitor (SKI-606) enhanced CEBPD abundance (Figure 3C). These results suggest that loss of Src activity contributes to stabilization of CEBPD protein upon metformin treatment.

### CEBPD participates in metformin-induced autophagy

**Figure 4:**
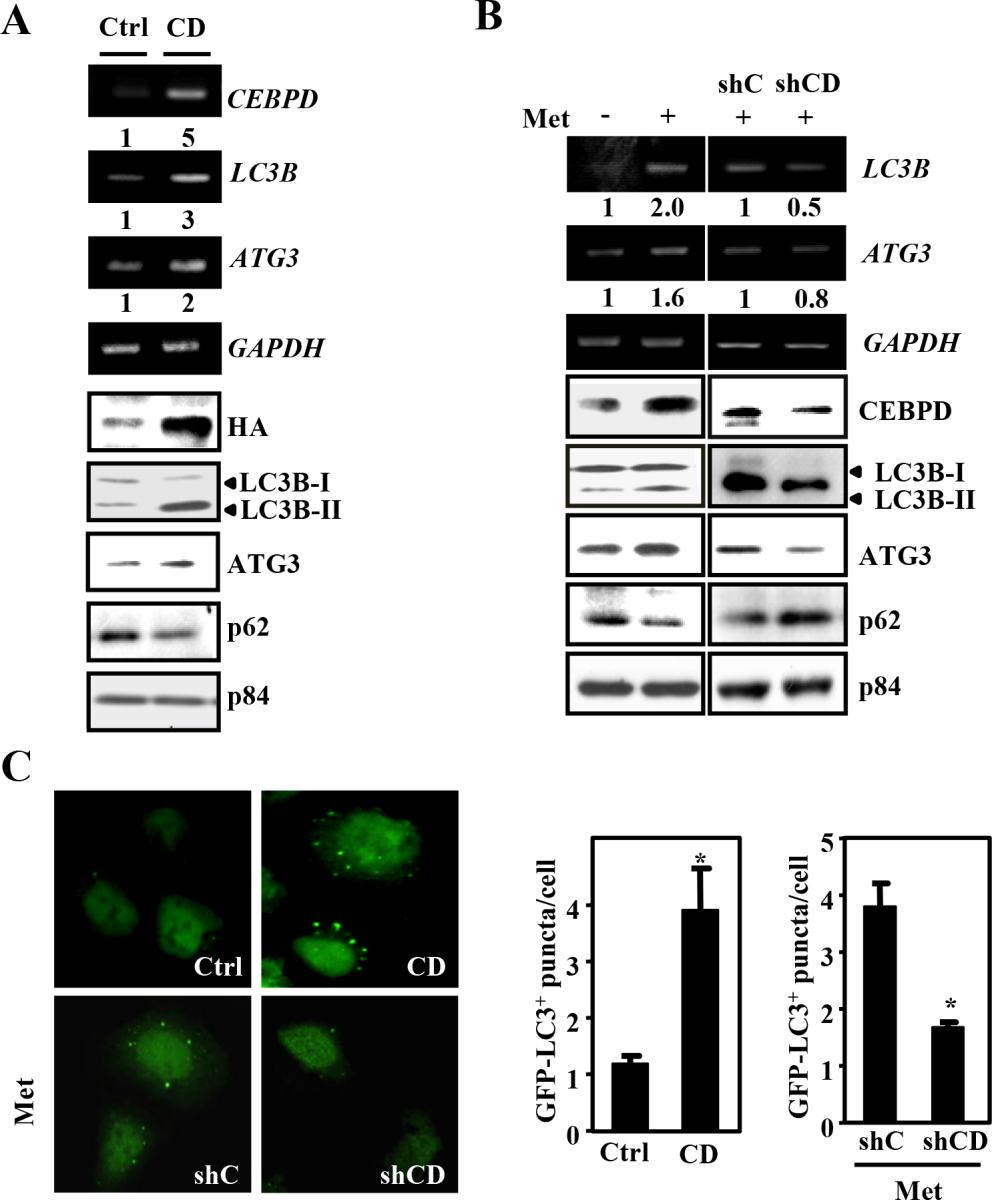
CEBPD is involved in metformin-induced autophagy (**A**) Huh7 cells were transfected with pcDNA3/HA (Ctrl) or pcDNA3/HA-CEBPD (CD) expression vectors or (**B**) infected with lentiviruses encoding shLacZ (shC) or shCEBPD (shCD) and then treated with or without metformin (Met, 5 mM). The lysates and total RNA were harvested for Western blot analyses and RT-PCR, respectively. (**C**) GFP-LC3B expression vectors were co-transfected with pcDNA3/HA (Ctrl) or pcDNA3/HA-CEBPD (CD) expression vectors into Huh7 cells (upper panel). Huh7 cells that were pre-infected with lentiviruses encoding shLacZ (shC) or shCEBPD (shCD) were transfected with GFP-LC3B expression vectors and then treated with Met (5 mM) for 6 h (lower panel). The numbers of LC3B puncta were evaluated under a fluorescence microscope.

As shown above, activation of both autophagy and CEBPD was observed in metformin-treated liver cancer cells. However, the association between the transcription factor CEBPD and autophagy activation remained unknown. Increase in CEBPD can enhance the LC3B-II/LC3B-I ratio and reduce p62 expression in Huh7 cells (Figure 4A). Moreover, loss of CEBPD reversed metformin-enhanced LC3B-II/LC3B-I ratio and metformin-reduced p62 expression (Figure 4B). In addition, increased numbers of LC3B puncta were observed in Huh7 cells exogenously expressing CEBPD (Figure 4C, upper panel), but the signal indicating LC3B puncta formation was reduced in CEBPD-knockdown Huh7 cells upon metformin treatment (Figure 4C, lower panel). These findings suggested that CEBPD is involved in metformin-induced autophagy.

### CEBPD activates *LC3B* and *ATG3* gene transcription in Huh7 cells

**Figure 5:**
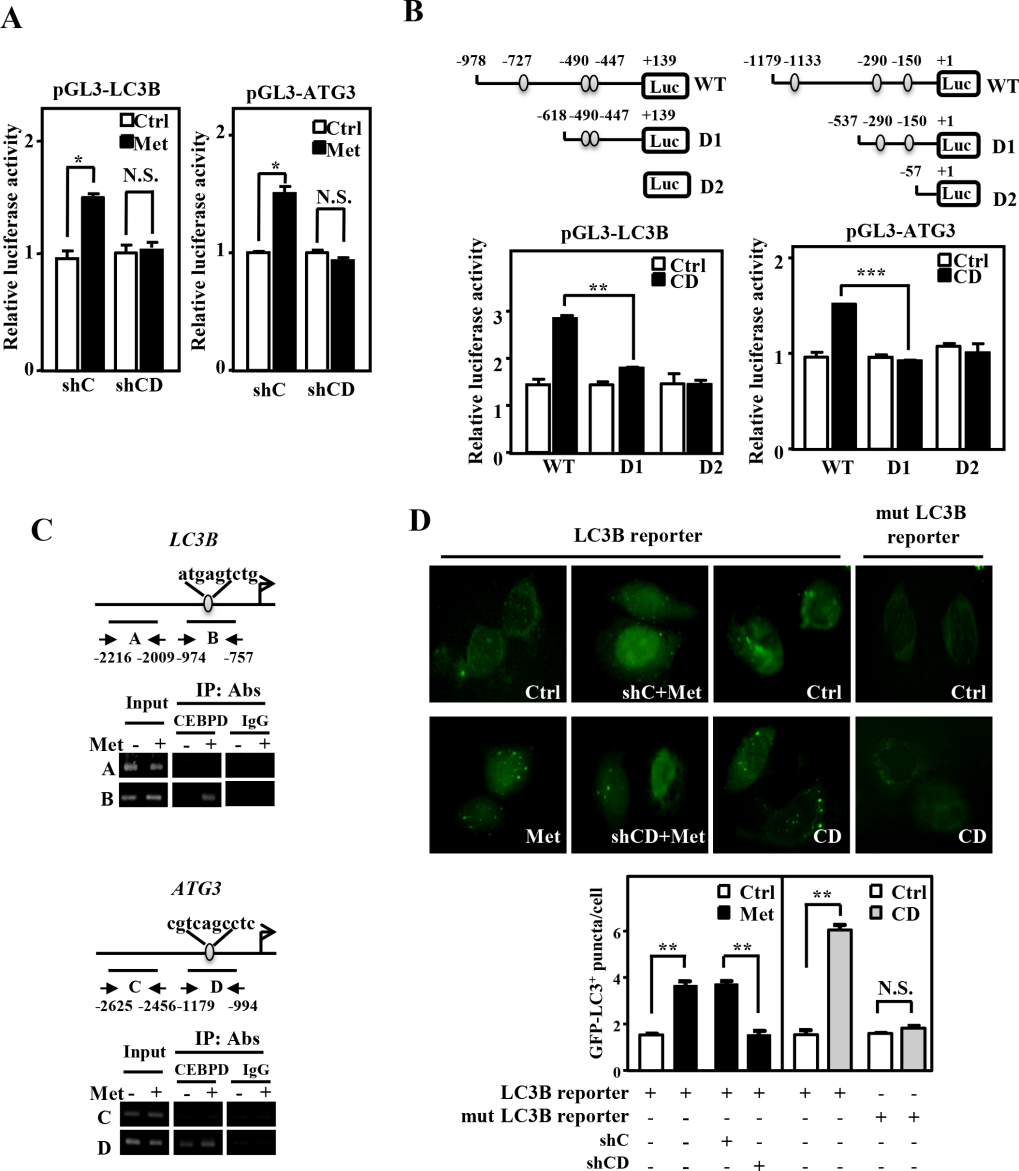
CEBPD activates *LC3B* and *ATG3* gene transcription and induces exogenous LC3B puncta formation (**A**) Huh7 cells that were pre-infected with lentiviruses encoding shLacZ (shC) or shCEBPD (shCD) were transfected with *LC3B* or *ATG3* reporters and then treated with or without metformin (Met, 5 mM). The lysates of experimental transfectants were harvested for luciferase assays. (**B**) Various 5′ serial deletion *LC3B* or *ATG3* reporters were co-transfected with pcDNA3/HA (Ctrl) or pcDNA3/HA-CEBPD (CD) expression vectors into Huh7 cells. The lysates of experimental transfectants were harvested for luciferase assays. (**C**) Huh7 cells were treated with or without Met (5 mM) for 6 h. As a positive control, PCR amplification was also performed using 1/20 of the input DNA from chromatin before the immunoprecipitation (IP) step. Chromatin was isolated from cells, and the IP step was performed using a CEBPD antibody. (**D**) Huh7 cells or Huh7 cells that were pre-infected with lentiviruses encoding shLacZ (shC) or shCEBPD (shCD) were transfected with LC3B promoter-driven GFP-LC3B expression vectors (LC3B reporter) and then treated with or without Met (5 mM) (Left panel). LC3B promoter-driven GFP-LC3B expression vectors (LC3B reporter) or CEBPD binding site-deleted LC3B promoter-driven GFP-LC3B expression vectors (mut LC3B reporter) were co-transfected with pcDNA3/HA (Ctrl) or pcDNA3/HA-CEBPD (CD) expression vectors into Huh7 cells (Right panel). The numbers of LC3B puncta were evaluated under a fluorescence microscope.

In addition to increase in the LC3B-II/LC3B-I ratio and reduction of p62 expression, the expression of ATG3 (the catalytic enzyme in LC3BI/II conversion) and total LC3B were also increased in CEBPD-overexpression Huh7 cells (Figure 4A). This observation led us to test whether CEBPD activated *LC3B* and *ATG3* gene transcription. *LC3B* and *ATG3* reporter activities were induced in metformin-treated Huh7 cells (Figure 5A). Meanwhile, CEBPD knockdown attenuated metformin-induced *LC3B* and *ATG3* reporter activities (Figure 5A). The result of a serial deletion reporter assay showed that potent CEBPD responsive regions were located at -978/-618 and -1179/-537 on the *LC3B* and *ATG3* gene promoters, respectively (Figure 5B). Furthermore, the *in vivo* DNA binding assay demonstrated that CEBPD bound to the promoter regions of *LC3B* and *ATG3* genes (Figure 5C). Next, a fused LC3B promoter-driven GFP-LC3B expression vector was constructed to assess whether CEBPD can induce LC3B puncta formation through activating *LC3B* transcription. The numbers of LC3B puncta increased and decreased following exogenous CEBPD increase and attenuation, respectively, with or without metformin treatment. Moreover, after deleting the CEBPD responsive region on the LC3B promoter, the numbers of LC3B puncta did not change following the exogenous increase of CEBPD (Figure 5D). Taken together, these results suggest that CEBPD activates the *LC3B* and *ATG3* gene transcription and increases LC3B puncta formation in a transcription-dependent manner.

### Combined treatment of metformin and rapamycin enhances the anticancer effects

A previous finding revealed that the activation of autophagy may enable residual cancer cells to resist chemotherapy and allow tumor relapse and progression [24]. However, excessive autophagy has been implicated in cell death [25]. Combined treatment to enhance the activity of autophagy is one choice for cancer therapy. Rapamycin, an mTOR inhibitor [26], has been suggested to serve an autophagy activator [27]. Western blot analyses showed that treatment with an AMPK inhibitor (compound C) suppressed metformin-induced AMPK phosphorylation and CEBPD expression, whereas rapamycin had no effect on AMPK phosphorylation and CEBPD expression in Huh7 cells (Supplementary Figure 5A). These results implied that metformin and rapamycin work via different pathways in autophagy activation. To check whether combined metformin and rapamycin treatment could enhance anticancer effects, cell viability and apoptosis were measured by MTT assay and flow cytometry PI staining, respectively. The MTT assay showed that combined metformin and rapamycin treatment reduced Huh7 cell viability further than individual treatment (Supplementary Figure 5B). Moreover, this combination significantly increased Huh7 cell apoptosis compared to metformin or rapamycin treatment alone (Supplementary Figure 5C). The results suggest that combined metformin and rapamycin treatment can enhance liver cancer cell death *in vitro*.

### Combination of metformin and rapamycin elicits stronger cytotoxicity in xenograft model

**Figure 6:**
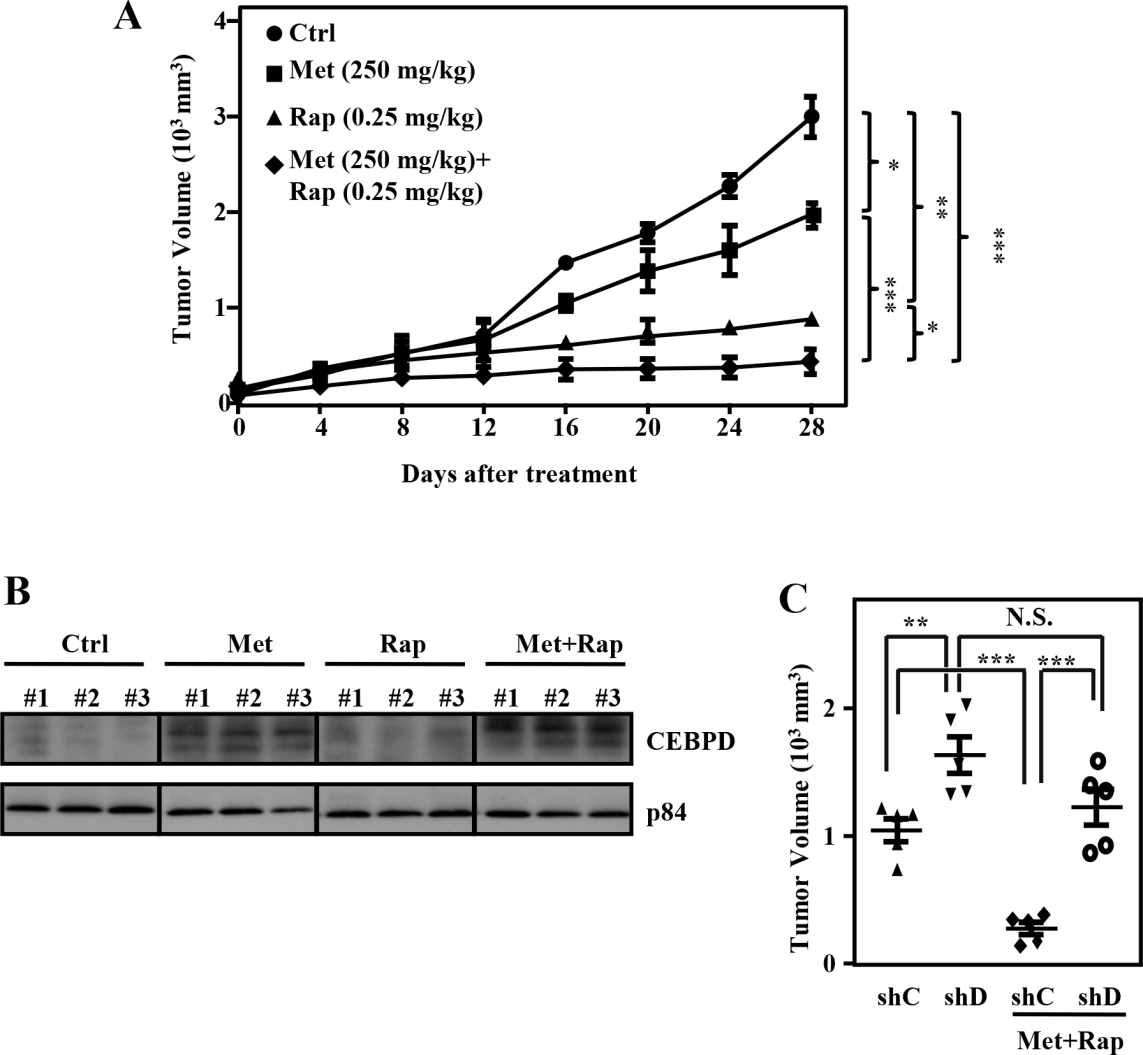
Combination of metformin and rapamycin elicits stronger cytotoxicity in xenograft model Huh7 cells were subcutaneously inoculated into NOD-SCID mice, followed by an intraperitoneal injection of vehicle, metformin (Met, 250 mg/kg/d), Rap (0.25 mg/kg/d), or Met (250 mg/kg/d) combined with Rap (0.25 mg/kg/d). (**A**) Tumor dimensions and animal weights were obtained at the indicated time points. (**B**) After 28-day treatment, the lysates extracted from these experimental xenograft tumors were collected and analyzed by Western blotting. (**C**) Huh7 cells were infected with lentiviruses to drive stable expression of either IPTG-inducible LacZ shRNA (shC) or IPTG-inducible CEBPD shRNA (shCD). Infected Huh7 cells were subcutaneously inoculated on the dorsal of 6-week-old NOD-SCID mice (*n* = 5) followed by intraperitoneal injection of 200 μl IPTG (0.53 mmol) every other day. Mice were then treated with or without Met (250 mg/kg/d) combined with Rap (0.25 mg/kg/d) via intraperitoneal injection. After 28-day treatment, mice were sacrificed and the tumor volume was measured.

We further evaluated the effect of combined treatment compared to individual treatment in human tumor xenograft mouse model. Rapamycin elicited side effects when used clinically [28]. We used relatively lower clinical doses of rapamycin combined with metformin to treat Huh7 cell xenografts in NOD/SCID mice. In the Huh7 cell xenograft model, treatment with metformin or rapamycin inhibited tumor growth compared with control. Moreover, combined metformin and rapamycin treatment produced significantly enhanced antitumor activity compared with single-agent treatment (Figure 6A). Importantly, mice tolerated the combined treatment well, as no body weight loss was observed during or after treatment (Supplementary Figure 6). Furthermore, the CEBPD protein level was examined in lysates extracted from these experimental xenograft tumors. The result showed that CEBPD abundance was induced with metformin treatment alone and combined treatment (Figure 6B). Moreover, comparing with their individual controls (Figure 6C, compare lane 1 with lane 2 and lane 3 with lane 4), loss of CEBPD significantly enhanced the growth of xenografted Huh7 tumor in NOD-SCID mice, suggesting that CEBPD indeed plays an antitumor role. Importantly, loss of CEBPD also attenuated the dual treatment-enhanced killing effect, further suggesting its response to metformin-induced killing effect (Figure 6C, compare lane 1 with lane 3 and lane 3 with lane 4).

## DISCUSSION

Autophagy is a conserved process by which cytoplasmic components are degraded by lysosomes. It is commonly seen as a cytoplasmic event, and nuclear events were not considered of primary importance for this process until now. In light of the recent findings on transcription factors EB (TFEB) [29], E2F1 [30], forkhead box O1 (FOXO1) [31], activating transcription factor 4 (ATF4) [32], CCAAT/enhancer-binding protein-β (C/EBPβ) [33] and C/EBP-homologous protein (CHOP) [34], we propose that a complex transcriptional network is involved in autophagy. The main function of most of the aforementioned factors is the transactivation of autophagy-related genes, including *LC3B*, allowing a sustained level of autophagy flux in the cells. However, the transcriptional activation of *ATG3*, which is indispensable for LC3BI/II conversion, is less characterized. Importantly, we identified CEBPD as a novel activator of *LC3B* and *ATG3* gene transcription. This is also the first study to demonstrate that transcriptional activation of autophagy-related genes contributes to the induction of apoptosis.

Src is a non-receptor tyrosine kinase that is upregulated in many types of cancer. The roles of Src in promoting tumor progression and metastasis have been well documented [35]. The most prominent and well-studied function of Src is its extensive interaction with transmembrane receptor tyrosine kinases (RTKs). Src interacts with epidermal growth factor receptor (EGFR), human epidermal growth factor receptor 2 (HER2 or ErbB2), platelet-derived growth factor receptor (PDGFR), insulin-like growth factor-1 receptor (IGF-1R) and c-Met/hepatocyte growth factor receptor (HGFR) [36]. Several studies have indicated that inhibition of EGFR tyrosine kinase or Src kinase can induce autophagy in cancer cells [37, 38]. Moreover, metformin inhibits the expression of the EGFR family in tamoxifen-resistant breast cancer cells [39]. These reports provide potential explanations why metformin could inhibit Src activity, as observed in this study (Figure 3B).

Epigenetic effects attenuated CEBPD expression in HCC [15, 16]. As mentioned above, loss of Src activity contributes to stabilization of CEBPD protein. This discovery indicates that the attenuation of CEBPD could also result from increased CEBPD protein degradation. However, this hypothesis requires further investigation. In addition, CEBPD has been suggested to serve as a potent tumor suppressor [40]. Strong CEBPD activation could strengthen the death of liver cancer cells [15]. CEBPD activation can be downregulated by Src kinase activity, as shown in this study. Therefore, the potential of Src inhibitor-containing combinatorial regimens can be tested in HCC following the rationale of activating and stabilizing CEBPD in cancer cells.

**Figure 7:**
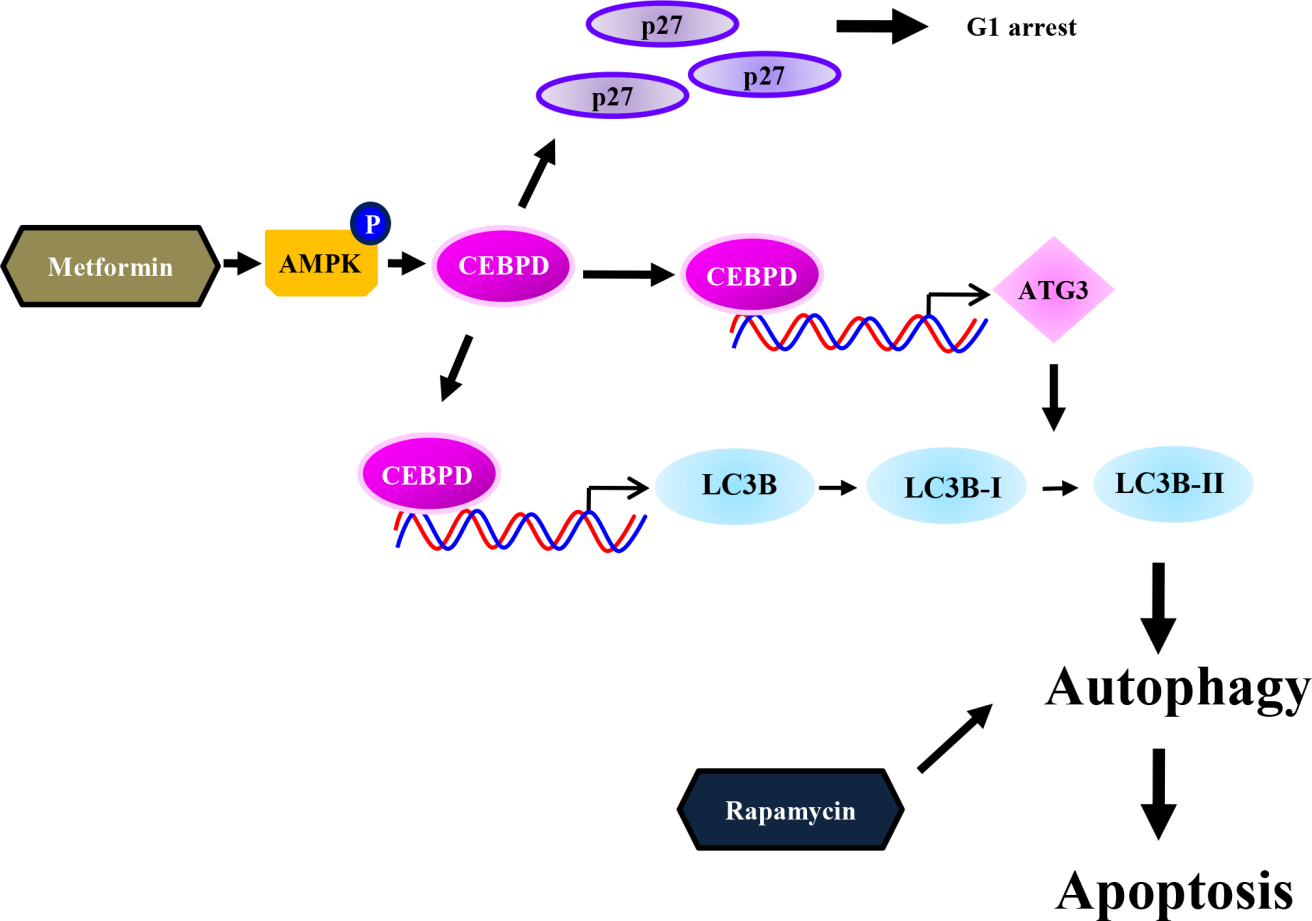
Schematic model of the molecular mechanism by which the combination of metformin and rapamycin enhances anticancer effects

Many cancers develop resistance to chemotherapy drugs under single treatment, which is a major factor in the failure of many forms of chemotherapy [41]. Combinatorial use of metformin and dichloroacetate (DCA) promotes B leukemic cell death [42], and suppresses the growth of ovarian cancer cells [43]. Moreover, metformin enhanced apoptosis induced by paclitaxel and cisplatin in endometrial cancer [44]. Metformin, as an AMPK activator, can also activate autophagy in cancer cells [12, 45–47]. AMPK is an essential mediator of the tumor suppressor LKB1 and could be suppressed in cancer cells [48]. In this study, we found that AMPK is involved in metformin-induced CEBPD expression. However, rapamycin had no effect on AMPK activation in myotubes [49], which is consistent with our findings. Therefore, combined metformin and rapamycin treatment can enhance autophagic cell death through AMPK-dependent and AMPK-independent pathways, respectively, and reduce the development of drug resistance in HCC (Figure 7).

Importantly, studies showed that metformin can attenuate the occurrence of HCC and reduce human tumor xenograft growth [50–53]. However, the potent mechanism in response to metformin in liver cancer cells remains an open question. In this study, we found that metformin can induce autophagy through CEBPD upregulation, and the combinatorial treatment of metformin and rapamycin can enhance autophagic cell death of HCC. In addition, CEBPD is thought to be a potent tumor suppressor, and its expression is downregulated in several cancers, including breast cancer [54], leukemia [55], cervical cancer [56], and hepatocellular carcinoma [57]. Several studies indicated that autophagy contributes to pro-apoptosis in breast cancer [58] and cervical cancer [59] and limits proliferation in leukemia [60]. Therefore, an issue whether the CEBPD-induced autophagy contributes to the death of breast cancer, leukemia and cervical cancer needs to be further dissected.

## MATERIALS AND METHODS

### Materials

TRIzol RNA extraction reagent, SuperScript^™^ III, Dulbecco's modified Eagle's medium, Opti-MEM medium, and caspase-3/7 green detection reagent were obtained from Invitrogen (Carlsbad, CA, USA). Fetal bovine serum was purchased from HyClone Laboratories (Logan, UT, USA). Metformin, rapamycin and chloroquine were purchased from Sigma (St. Louis, MO, USA). Compound C (AMPK inhibitor) was purchased from Calbiochem (San Diego, CA, USA). SKI-606 was purchased from Selleck Chemicals (Houston, TX, USA). Antibodies against CEBPD, cyclin D1, AMPK, p-AMPK and β-actin were purchased from Santa Cruz Biotechnology (Santa Cruz, CA, USA). Antibodies against LC3B, p27, and p84 were purchased from GeneTex (Irvine, CA, USA). Antibodies against ATG3 and p62 were purchased from Cell Signaling Technology (Beverly, MA, USA). Antibodies against HA, Src and p-Src were purchased from Covance (Berkeley, CA, USA). Antibodies against α-tubulin and mouse IgG were purchased from Sigma.

### Cell culture

The human hepatocellular carcinoma cell lines Huh7, HepG2 and Hep3B were maintained in Dulbecco's modified Eagle's medium (DMEM) supplemented with 10% fetal bovine serum (FBS), 100 μg/ml streptomycin, and 100 units/ml penicillin at 37°C and 5% CO_2_.

### Lentiviral shRNA knockdown

The virus was produced from Phoenix Ampho cells using Mirus Bio TransIT-2020 and cotransfected with various short hairpin RNA (shRNA) expression vectors in combination with pMD2.G and psPAX2 vectors and the pLKO.1-shRNA expression vectors. The short interfering RNA sequences targeting LacZ, CEBPD and LC3B were subcloned into the lentiviral expression vector pLKO.1. The short interfering RNA sequences are as follows: shLacZ (shZ): 5′-CCGGTG TTCGCATTATCCGAACCATCTCGAGATGGTTCGGA TAATGCGAACATTTTTG-3′; shCEBPD (shD): 5-CCG GGCCGACCTCTTCAACAGCAATCTCGAGATTGCT GTTGAAGAGGTCGGCTTTTT-3; shLC3B (shL): 5-CCG GCGCTTACAGCTCAATGCTAATCTCGAGATTAGCA TTGAGCTGTAAGCGTTTTTTG-3. The expression vectors and shRNAs were obtained from the National RNAi Core Facility located at the Genomic Research Center of Institute of Molecular Biology, Academia Sinica, Taiwan.

### Plasmid transfection and reporter assays

Several varieties of human CEBPD reporters were constructed in our lab [61]. The reporters bearing different fragments of the human ATG3 and LC3B promoters were generated by PCR with genomic DNA as the template. The primers for the PCR reaction are as follows: ATG3 promoter (WT) 5′-CTCACTGCAACCCCTACCTA-3′ and 5′-GAAGCGGACGCACACGC-3′; ATG3 promoter (D1) 5′-CCTTCTAGTCACACATGCGC-3′ and 5′- GAA GCGGACGCACACGC-3′; ATG3 promoter (D2) 5′-GAA GCAAAGCGAGGACAGAC′ and 5′- GAAGCGGACGC ACACGC-3′; LC3B promoter (WT) 5′-CGGGAGGC TGAGGCAGGAGGATCC-3′ and 5′-CGATAGCCACTT CCCTTGTATCTC-3′; LC3B promoter (D1) 5′-AGTCGC CACAGACGACCTAA-3′ and 5′-CGATAGCCACTTCC CTTGTATCTC-3′. After verification by sequencing, the PCR products were cloned into the multiple cloning sites of pGL3-basic vector. These reporters were transfected into Huh7 cells by Turbofect according to the manufacturer's suggestions. Transfectants were cultured in complete medium with or without treatment for 6 h. Luciferase activity was measured in the lysates of transfectants.

### Reverse transcription (RT)-PCR

We isolated total RNA from stimulated Huh7 cells and used CEBPD-, ATG3-, and LC3B-specific primers for analysis. Glyceraldehyde-3-phosphate dehydrogenase (GAPDH) primers were used as a control. The specific primers used were as follows: human CEBPD: 5′-AGCGCAACAACATCGCCGTG-3′ and 5′-GTCGGGTCTGAGGTATGGGTC-3′; ATG3: 5′-CA ACGGCAGCCTTTAACAGT-3′ and 5′-CAAGTTCTCCC CCTCCTTCT-3′; LC3B: 5′-AGCAGCATCCAACCAAA ATC-3′ and 5′-CTGTGTCCGTTCACCAACAG-3′; human GAPDH: 5′- CCATCACCATCTTCCAGGAG-3′ and 5′- CCTGCTTCACCACCTTCTTG-3′. Amounts of PCR product were determined using the IS-1000 digital imaging system (Alpha Innotech, San Leandro, CA, USA).

### Quantitative real time polymerase chain reaction (Q-PCR)

Real-time PCR was performed using KAPA SYBR FAST qPCR Master Mix and the CFX95^™^ Real-Time PCR Detection machine. Primer sequences were as follows: CEBPD: 5′-GCCATGTACGACGACGAGAG-3′ and 5′-TGTGATTGCTGTTGAAGAGGTC-3′; GAPDH: 5′-C CACCCAGAAGACTGTGGAT-3′ and 5′-TTCAGCTCA GGGATGACCTT-3′. Q-PCR was conducted with the following amplification conditions: 1 cycle of 95°C for 10 min, 39 cycles of 95°C for 5 sec, 60°C for 10 sec and 72°C for 15 sec. Finally, a melting curve was performed to check for the presence of a single product from each reaction. Expression levels of the genes of interest were then compared to expression of GAPDH.

### Chromatin immunoprecipitation (ChIP)

ChIP assay was conducted as described by Ju-Ming Wang and colleagues [62]. Briefly, the sheared chromatin fragments were immunoprecipitated with antibodies specific to CEBPD or control mouse IgG at 4°C overnight. After dissociating the DNA from immunoprecipitated chromatin, the DNA was amplified by PCR with two pairs of specific primers: ATG3 (F) 5′-CTCACTGCAACCCCTACCTA-3′ and ATG3 (R) 5′-AAAGTGCTGGGATTACAGG-3′; the negative control for ATG3 (F) 5′-GGGACATGCGGGCAACAATA-3′ and the negative control for ATG3 (R) 5′-CACTCTCCGAG AAACACCCA-3′; LC3B (F) 5′-GAGACACATTGGCC ACAGGC-3′ and LC3B (R) 5′-AGTCGCCACAGACGA CCTAA-3′; the negative control for LC3B (F) 5′-CCAC CTGCACAAATGCACCG-3′ and the negative control for LC3B (R) 5′-TGTATTCCCAGCTACTGGGG-3′. The PCR conditions were as follows: 1 cycle of 3 min at 94°C; 36 cycles of 94°C for 30 sec, 56°C for 30 sec, 72°C for 40 sec, and 1 cycle of 10 min at 72°C.

### Western blot analysis

Cell lysates were prepared from control and chemically treated cells. Briefly, cells were lysed in modified RIPA buffer [50 mM Tris-HCl (pH 7.4), 150 mM NaCl, 1 mM EDTA, 1% Nonidet P-40, 0.25% sodium deoxycholate, 1 mM DTT, 1 mM phenylmethylsulfonyl fluoride, 1 μg/ml aprotinin and 1 μg/ml of leupeptin] to be analyzed. Following lysis, the lysates were resolved on an SDS-containing 10% polyacrylamide gel, transferred to polyvinylidene difluoride nylon membrane, and probed with specific antibodies at 4°C overnight. The specific bands were detected by horseradish peroxidase-conjugated antibody and revealed by an enhanced chemiluminescence (ECL) Western blot system from Pierce (Rockford, IL).

### mRNA half-life assay

Huh7 cells were treated with or without metformin for 2 h. Then, 1 μg/ml actinomycin D was added to block newly synthesized RNA. Total RNA was isolated at the indicated times and examined by qPCR.

### Protein stability

Huh7 cells were treated with or without metformin for 2 h. Then, 10 μg/ml cycloheximide was added to block newly synthesized protein. Cell lysates were isolated at indicated times and analyzed by Western blotting assay.

### Cell viability

Huh7 cells were seeded 5 × 10^3^ cells/ well in 96-well plates. Cells were treated with various concentrations (0, 5, 10 and 20 mM) of metformin for 48 h or with the combination of 5 mM metformin and 10 μM rapamycin for 48 h. The experimental cells were incubated with diluted MTT reagent (3-(4,5-dimethylthiazol-2-yl)-2,5-diphenyltetrazolium bromide) at 37°C for 3.5 h. The samples were then measured spectrophotometrically at 595 nm by an ELISA plate reader.

### Flow cytometry analysis

Typical cell cycle histograms were analyzed with flow cytometry after treatment with metformin alone or combined with rapamycin for the indicated times. Apoptotic cell death of Huh7 cells was determined by analyzing the sub-G1 populations. After chemical treatment, cells were resuspended in PBS and fixed in ice-cold 75% ethanol at −20°C. Later, cell pellets were collected by centrifugation and resuspended in propidium iodide solution (0.1% Triton X-100 in PBS, 0.2 mg/ml RNase A and 20 μg/ml propidium iodide) at room temperature for 1 h. Fluorescence emitted from propidium iodide-DNA complexes was quantified after excitation of the fluorescent dye by FACScan cytometry (CellLab Quanta^™^ SC, *Beckman Coulter*).

### Caspase-3/7 activity assay

After treatment with or without metformin for 48 h, cells were labeled with 2 μM CellEvent^™^ caspase-3/7 green detection reagent in complete medium for 1 h at 37°C in the dark. The fluorogenic response was detected on a Fluoroskan Ascent^™^ FL Microplate Fluorometer and Luminometer (ThermoLabsystem) with an absorption/emission of 485/538 nm.

### Fluorescence microscopy

The pEGFP-LC3 plasmid was a gift obtained from Dr. Tamotsu Yahsimori and Noboru Mizushima [63]. A fused LC3B promoter-driven GFP-LC3B expression vector was generated by replacing the CMV promoter region with LC3B promoter fragment from −978 to +139 bp obtained by PCR and the primers were described as follows: LC3B/AseI-978: 5′-GTTATTAATAGTCGGGAGGCTG AGGCAGGAGGAT-3′, LC3B/AgeI+139: 5′-GCTACC GGTCGCCGATAGCCACTTCCCTTGTATC-3′. Huh7 cells transfected with GFP-LC3B or its derived plasmids were grown on glass coverslips, co-transfected with HA/HA-CEBPD or treated with metformin (5 mM) for 6 h, and then examined under a fluorescence microscope. Images shown are representative of three independent experiments. The fold changes of the average numbers of puncta per positive cells were calculated with 50 individual cells.

### Animal studies

Male, six-week-old NOD/SCID mice were obtained from the Laboratory Animal Center of National Cheng Kung University, Tainan, Taiwan. Huh7 cells (2 × 10^6^) in 0.2 ml PBS were inoculated subcutaneously into the right flank of the mice. After 10 days, when macroscopic tumors (50–100 mm^3^) had formed, animals (*n* = 5 per group) were placed randomly into four groups as follows: (1) the control group, which received identical volumes of vehicle (DMSO); (2) the metformin treatment group, which was treated with 250 mg/kg/day metformin; (3) the rapamycin treatment group, which was treated with rapamycin at doses of 0.25 mg/kg/day; and (4) the combined treatment group, which was injected with metformin combined with rapamycin. Treatment was given to all groups intraperitoneally every day for four weeks. Animal weight and tumor dimensions were measured every four days with calipers, and tumor volumes were estimated using two-dimensional measurements of length and width and were calculated with the formula: [*l* × (*w*)^2^] × 0.52, where *l* is length and *w* is width.

### Statistical analysis

All experiments were repeated at least three times, and data were analyzed for statistical significance by two-tailed unpaired Student's *t* test using Prism 5 software. The data were expressed as the means ± SEM. Differences were considered statistically significant when indicated by asterisks. Statistical significance was accepted for *P* < 0.05 (*), *P* < 0.01 (**), or *P* < 0.001 (***).

## SUPPLEMENTARY MATERIALS FIGURES


