# Supplementary material for: Metformin promotes apoptosis in hepatocellular carcinoma through the CEBPD-induced autophagy pathway
Source: Oncotarget. 2017 Jan 13;8(8):13832–45. doi: 10.18632/oncotarget.14640 (PMC5355142; doi:10.18632/oncotarget.14640)
Supplement: Supplementary file 1 [file oncotarget-08-13832-s001.pdf]

Metformin promotes apoptosis in hepatocellular carcinoma through the CEBPD-induced autophagy pathway

Supplementary Materials

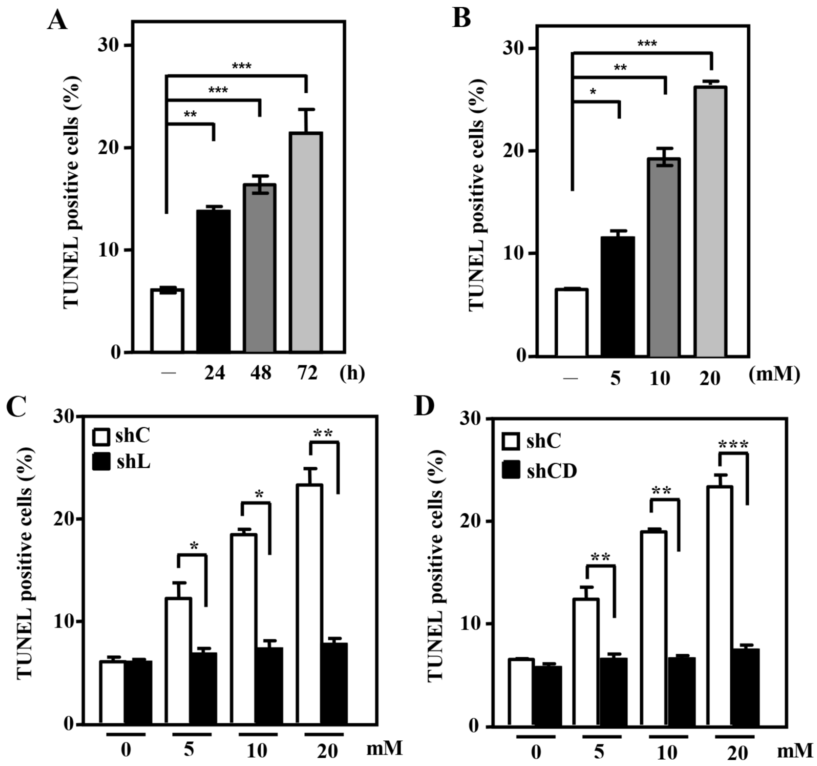

Supplementary Figure 1: Metformin induces cell apoptosis in Huh7 cells. (**A**) Huh7 cells were treated with metformin (Met, 5 mM) for 24, 48 and 72 h or (**B**) treated with Met (5, 10 and 20 mM) for 48 h. Experimental cells were collected at the indicated concentrations and time points and analyzed by TUNEL assay. (**C**) Huh7 cells were infected with lentiviruses encoding shLacZ (shC) or shLC3B (shL) for 3 days. The apoptotic activity of infected experimental cells was measured by TUNEL assays after 48 h of Met treatment at the indicated concentrations. (**D**) Huh7 cells were infected with lentiviruses encoding shLacZ (shC) or shCEBPD (shCD) for 3 days. The apoptotic activity of infected experimental cells was measured by TUNEL assays after 48 h of Met treatment at the indicated concentrations. Quantification of the data was analyzed by ImageJ.

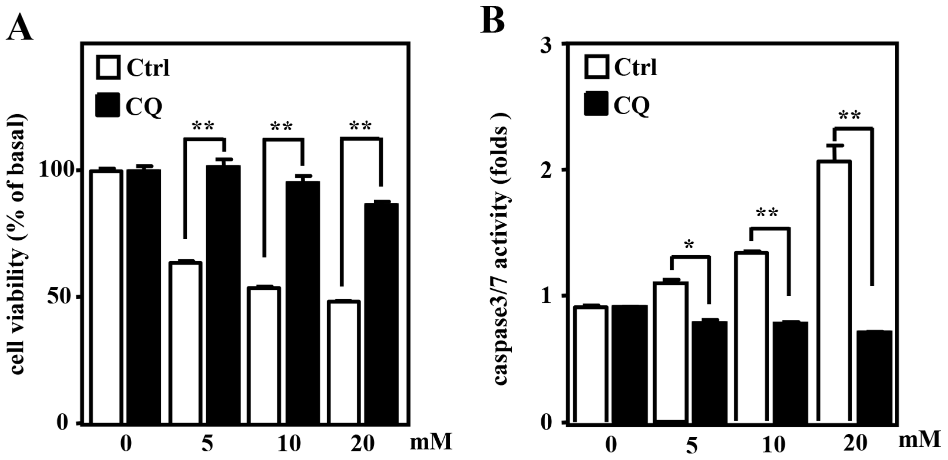

Supplementary Figure 2: Inhibition of autophagy by chloroquine reverses metformin-mediated anticancer effects. (**A**) Huh7 cells were pretreated with or without chloroquine (CQ, 10 μM) for 30 min and then treated with metformin (Met) at the indicated concentrations (5, 10 and 20 mM) for 48 h. The cell viability of experimental cells was measured by MTT assays. (**B**) The caspase-3/7 activity of the experimental cells was detected using *CellEvent caspase-3/7 green detection reagent*.

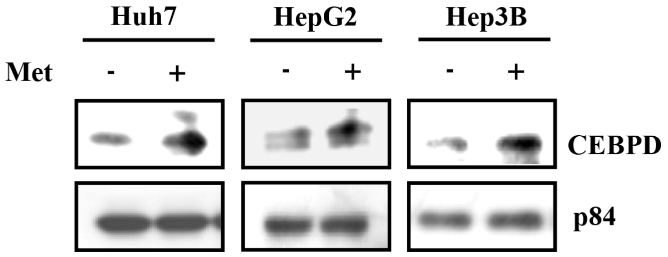

Supplementary Figure 3: CEBPD is upregulated in metformin-treated liver cancer cells. Three of HCC cell lines (Huh7, HepG2, and Hep3B) were treated with or without metformin (Met, 5 mM) for 2 h, and the lysates were harvested for Western blot analyses.

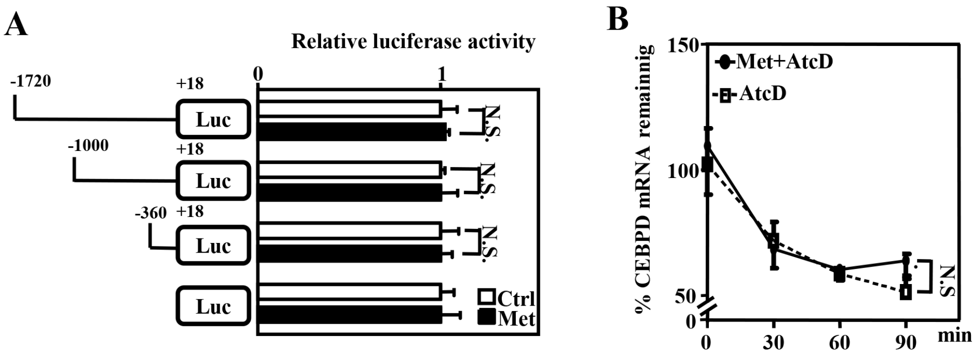

Supplementary Figure 4: Metformin has no effect on the regulation of CEBPD transcription. (**A**) Huh7 cells were transfected with various *CEBPD* 5' serial deletion reporters with or without metformin (Met, 5 mM). The lysates of the transfected cells were harvested for luciferase assays. (**B**) Huh7 cells were pretreated with or without Met (5 mM) for 2 h and then treated with actinomycin D (ActD, 1 μg/ml) to inhibit *de novo* synthetic transcripts. Total RNA was extracted at indicated time points, and the remaining transcripts were analyzed by real-time PCR assays.

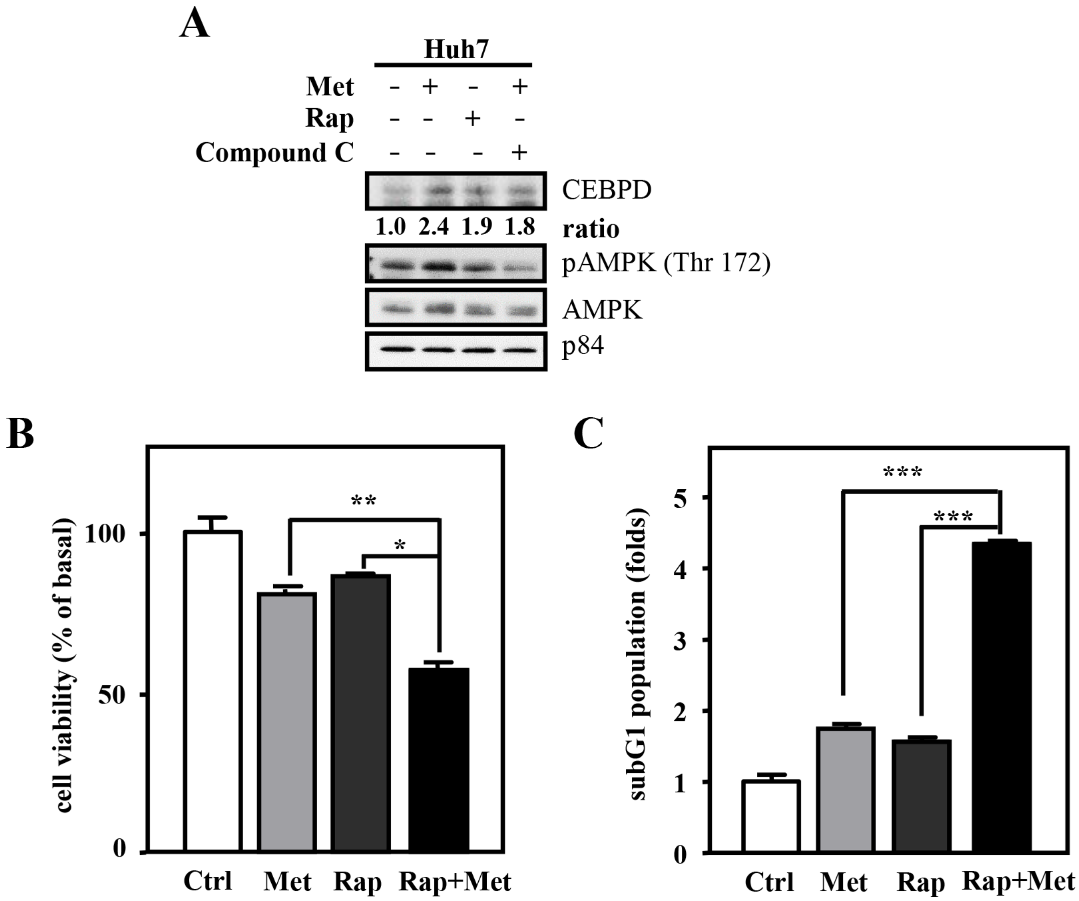

Supplementary Figure 5: Metformin and rapamycin work via different molecular mechanisms to enhance liver cancer cell death. (**A**) Huh7 cells were pre-treated with or without compound C (10 μM) for 30 min and then treated with metformin (Met, 5 mM) or rapamycin (Rap, 10 nM) for an additional 6 h. The lysates were harvested for Western blot analyses. (**B**) Huh7 cells were treated with Met (5 mM) or Rap (10 nM) alone or with the combination of Met and Rap for 48 h. The cell viability was measured by MTT assays. (**C**) Huh7 cells were treated with Met (5 mM) or Rap (10 nM) alone or with the combination of Met and Rap for 48 h. The apoptotic activity of experimental cells was analyzed by flow cytometry PI staining.

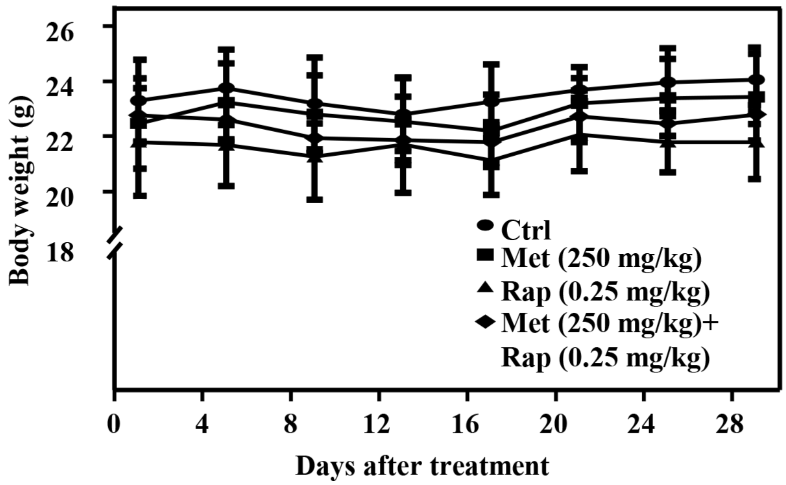

Supplementary Figure 6: There is no body weight loss during or after treatment. Animal weights were obtained at the indicated time points.
